# A cross sectional study on the different domains of frailty for independent living older adults

**DOI:** 10.1186/s12877-019-1077-3

**Published:** 2019-03-01

**Authors:** Didi Verver, Hanneke Merten, Carolien de Blok, Cordula Wagner

**Affiliations:** 1 Amsterdam Public Health research institute, Department of public and occupational health, VU University Medical Center, Van der Boechorststraat 7, 1081 BT Amsterdam, the Netherlands; 2 Rekenkamer Metropool Amsterdam, Weesperstraat 105a, 1000 AE Amsterdam, the Netherlands; 3 The Netherlands Institute for Health Services Research (NIVEL), Otterstraat 118-124, 3513 CR Utrecht, the Netherlands

**Keywords:** Frailty, Older adults, Independent living, Ageing in place, Policy

## Abstract

**Background:**

With the ageing population, there is a stronger focus on supporting older people to live independently as long as possible. One of the important factors to take into consideration for independent living older adults is frailty. This manuscript aims to provide insight into the relation between the different domains of frailty (physical, social and psychological or a combination), health outcomes and wellbeing aspects for independent living older adults.

**Methods:**

This cross sectional community-based study included independent living older adults of 65 years and over who are member of a welfare organisation*.* The questionnaire contained items on background characteristics, health, quality of life, frailty (Tilburg Frailty Indicator), activities and loneliness. A multivariate analysis, one Way ANOVA’s and chi-square tests with post-hoc analyses were used to identify significant differences between the following outcomes: Age, gender, marital status, living situation, income, health perception, number of conditions, activities of daily living, home care and informal care, Quality of life, loneliness, going outside, meeting people and the different domains of frailty.

**Results:**

1768 (35.1%) participants completed the questionnaire. 68.9% of the respondents was frail on one or multiple domains and 51.6% of the respondents was frail based on the total score on the TFI. Social frailty (18.4%) was most often present followed by 10.3% for frailty on all three domains of the TFI. All variables tested, except for income, showed significant differences between the different domains of frailty.

**Conclusion:**

Distinguishing the different domains of frailty provides information about the older adult’s needs which is valuable for policymakers and care providers, to anticipate to the increasing number of independent living older adults and deliver them tailored care and support to contribute to their independent living situation and wellbeing.

**Electronic supplementary material:**

The online version of this article (10.1186/s12877-019-1077-3) contains supplementary material, which is available to authorized users.

## Background

The population is ageing worldwide and people live longer in good health [1, 2]. Also, the proportion of people over 80 years old is rising [3]. These changes may threaten the sustainability of welfare systems of western countries. As a result, many western countries adopted some form of,‘ageing in place’ policy [4, 5], aiming to prolong the independent living status of older adults at home instead of institutionalisation. A recent study in the USA found that support for aging in place for the home care population can measurably delay nursing home admission by 8 months [6]. This policy is mainly performed to limit health and social care costs, however, older people also mainly prefer to age in place [7, 8].

This is also the case for the Netherlands. The Dutch government has decided to focus on enhancing self-sufficiency and independent living as long as possible, which leads to more independent living frail older adults, and as a consequence potential complex care situations in their home setting [9]. In addition, many of the responsibilities to support these independent living older adults have been shifted to the municipalities since January 2015. An important assumption for this decision is that municipalities should be more capable to address needs and wishes of independent living people on the local level, and they are better able to organize the needed care and support [10]. The changes in life expectancy, living situations and responsibilities make it important for municipalities, and other policy making agencies, to anticipate to the health and wellbeing situation of their frail older inhabitants, so they can offer sufficient care and support.

Frail older adults have an increased risk for negative health outcomes like mortality, morbidity and admission to a hospital or nursing home [11]. These risks contribute to the rising healthcare costs and have a negative impact on the quality of life of the older adults and their potential to live independently [12]. In total, the Netherlands had 16,574.989 inhabitants of which 2538.328 were 65 years or older in 2010 [13] There were 690.000 frail adults of 65 years and older in the Netherlands in 2010 according to the Netherlands Institute for Social Research (SCP), a proportion of 27%. Most of them, between 500.000 and 600.000, lived independently, the remaining 90.000–190.000 lived in an institution. The SCP expects an increase in the number of frail older adults in the Netherlands between 2010 and 2030 from almost 690.000 up to more than one million [14], which will cause an increase in the care and support needed for this group to enable them to live independently.

A number of frailty assessment tools have been developed in the past years, the majority of these tools include only items on physical frailty characteristics [15]. The Frailty Index [16], the Groningen Frailty Indicator [17], the Tilburg Frailty Indicator (TFI) [18] and the Edmonton Frail Scale [19] are examples of tools which do include the different domains. According to a recent study by Roppolo et al.(2015) different instruments based on different conceptualizations of frailty detect different individuals as frail. However, they state that a multidimensional tool would be better able to detect individuals at risk for negative health outcomes [20]. Also a study by Khezrian endorses frailty tools which provide a composite measure of frailty [21]. The definition of frailty, as presented by the SCP [14], also emphasizes the focus on the accumulation of problems on the different domains, the physical, psychological and social domain are all likely to be key issues. Complex interactions in these domains may contribute to the degree of frailty in an individual. To enable integrated and seamless care it is important to pay attention to the individual as a whole and take all three domains into account. This also fits the movement of positive health by Machteld Huber [22] and the definition of the World Health Organization (WHO), which defines health as ‘a state of complete physical, mental, and social well-being and not merely the absence of disease or infirmity’ [23]. This paper adds to the knowledge of which variables may contribute to the different domains of frailty, since the association between lifestyle factors and frailty is not a well-studied topic [24].

The increase of independent living frail older adults and changes in government and municipality responsibilities for supporting them, make it important for municipalities, policymakers and care and welfare providers to assess important characteristics, health outcomes and wellbeing aspects of this population to offer them tailored support in care and wellbeing within the home situation. This paper aims to provide insight into the relation between these characteristics and different domains of frailty.

## Methods

### Design and setting

This manuscript displays and discusses the baseline data of a quantitative evaluation study ‘*Home Sweet Home*’. The Home Sweet Home project was executed by ‘WonenPlus’, a collective name for multiple welfare organizations which deliver support to independent living older adults in different regions of the province of North-Holland in the Netherlands. This project was financed through the National Care for the Elderly Programme of The Netherlands Organization for Health Research and Development (ZonMw), which was designed to improve care for older people with complex care needs. The Amsterdam Public Health research institute /VUmc evaluated this project. Activities of the WonenPlus organizations aim to contribute to the wellbeing and support of independent living older adults and other people with mental or physical disabilities. The study aimed to compare the standard reactive method to an adjusted method which proactively approached independent living older adults to offer them support. With the reactive method, the organization provided help and support only when the older adult asked for support. With the proactive method employees and volunteers reached out to the older adults, who already were a member of the ‘WonenPlus organization’, and offered their help and support.

Data for the current study on the baseline measurement was collected between January 2011 and April 2012 using paper-based quantitative questionnaires. Questions from the ‘The Older Persons and Informal Caregivers Survey Minimum DataSet’ (TOPICS-MDS) [25] were included, since this was an obliged component for funding. These items were added in the questionnaire so one questionnaire could be used. The TOPICS-MDS contains questions about health, multi morbidity, quality of life and care usage. The aspects of health of interest for this manuscript were; health perception, number of conditions and ADL problems. Next to the TOPICS-MDS, complementary questions were added to the questionnaire about social contacts, loneliness [26, 27], frailty [18] and other topics. The questionnaire was developed for this study, using validated questionnaires and additional questions. See Additional file 1 for the additional questions.

Owing to privacy reasons, questionnaires were sent to potential participants, who were a member of the WonenPlus organization, by the WonenPlus organizations and they collected the data. After deleting all possible participant identifiers by the WonenPlus organizations the anonymous questionnaires were handed over to the researchers.

### Inclusion criteria

In this study, we defined independent living as follows: older adults who either lived alone, with their spouse or other relatives in their own home. Older adults living in sheltered accommodation or housing designed for older people with on-call staff were excluded from the definition independent living. Participants had to be 65 years or older, able to give informed consent, live independently and be a member of a WonenPlus organization in a rural or urban environment. All members of 65 years and older registered in seven different WonenPlus-client databases were asked to participate in the study, which were 5026 older adults in total.

At the baseline measurement the response for participation was 35.1%; 1768 older adults completed the questionnaire and gave written informed consent. 1085 (61.4%) completed the questionnaire independently and 604 (34,2%) respondents had some kind of help with filling out the questionnaire, of 75 (4.5%) respondents this item was missing. Reasons for non-response are not available, since data was collected by the Wonenplus organizations and follow-up on the non-response by the researchers was not possible due to privacy reasons.

### Frailty, Tilburg Frailty Indicator (TFI)

The SCP (2011) describes frailty as follows: “A process of an accumulation of physical, psychological and/or social deficits in functioning which increases the chance of adverse health outcomes (functional disabilities, admission to an institution, death)” [14]. The TFI of Gobbens et al. (2010) is an instrument to operationalize and measure frailty. This instrument distinguishes different domains of frailty, namely; physical, psychological and social [18, 28]. In this study the TFI was used to assess frailty, using 15 items concerning frailty. Scores on the different domains were counted as follows: a score of 4 or higher on the eight items of physical frailty was assessed as physically frail. A score of two or higher on the four items of psychological frailty was assessed as psychologically frail. A score of two or higher on the three items of social frailty were assessed as socially frail [14]. The total score on the TFI was calculated to determine overall frailty; a score of five or higher was classified as being frail [18]. The psychometric properties of the TFI have been established by the developers of this questionnaire, the internal consistency was measured as Cronbach’s α = 0.73, indicating moderate internal consistency [18, 29]. In the current study, it was decided that when an item on the TFI was missing, it was counted as ‘0’, so it would not contribute to the degree of frailty, but overall scores on the different domains and a total score could be calculated for all respondents. This decision was taken since the number of missing values for items three up to eight in the TFI was extensive, but the respondents appeared to only have answered the question when their answer was ‘yes’ (contributing towards frailty) and left it blank when their answer was ‘no’ (not contributing to frailty). The number of missing values was analysed using frequencies and a missing value analysis on different items was performed. Missing values were imputed and the imputed data was compared to the original data. These steps will be further explained in the section on the statistical analyses.

### Health aspects

The following aspects of health were analysed:

- *Health perception* is analysed in three categories; excellent/very good, good and reasonable/bad
  *Number of conditions;* ranging from zero up to eleven. The participants were asked to check the box when they had one or more of the following diseases or conditions in the past 12 months: diabetes, stroke, heart failure, cancer, asthma or other lung problems, incontinence, joint wear, depression, anxiety or panic disorder, dementia, hearing problems and vision problems.
- *Activities of daily living (ADL), KATZ*
- To assess the level of both basic and instrumental activities of daily living, the KATZ-15, also known as the Modified Katz ADL, was used. This instrument was established as internally consistent (KR-20 = 0.8) and strongly associated with quality of life measures [30]. The instrument includes 15 items and a total score on the 15 items was calculated, the higher the score, the more problems with the ADL are present. When two or more items were missing, the respondents were left out of the analyses.
- *Home care and informal care*
- Usage of home care and informal care were analysed using the following questions at which the respondents could answer ‘yes’ or ‘no’:
  -
*‘Do you use home care? For example, a district nurse or help at home‘*
  -
*‘Do you have someone who voluntarily helps or supports you for a longer period of time? (informal care provider)’*

### Wellbeing aspects

The following aspects of wellbeing were analysed:

- *Quality of life,* the respondents graded their quality of life and compared to a year ago (much better, little better, the same, little worse, much worse).
- *Loneliness,* loneliness was assessed using the loneliness scale of de Jong-Gierveld with a 3-point likert scale and a reliability of Cronbach’s α = 0.80 up to 0.90 [27]. The loneliness scale has 11 items and a total score on the 11 items was calculated, analyses were done with the total score as continuous variable. When two or more items were missing, the respondents were left out of the analyses. To create the imputed data file loneliness was used as a categorical variable to lower the number of parameters; not lonely (score 0, 1 or 2), moderate lonely (score 3 through 8), severe lonely (score 9 or 10), and very severe lonely (score 11).

*Going outside;* daily, few times per week, few times per month, less than once a month

*Meeting the children, grandchildren, family, friends and neighbours;* every week, once or twice per month, less/seldom, never, not applicable

### Statistical analysis

IBM SPSS (SPSS Inc., Chicago, Illinois) version 22.0 was used to analyse the data. Descriptive statistics were used to calculate frequencies on the different domains of frailty. Because of the large amount of missing data for the TFI items 3,4,5,6,7 and 8 it was decided to conduct a missing value analysis, using Multiple Imputation in SPSS, to analyse the potential overlap in answers on related variables. Little’s Missing Completely at Random (MCAR) Test was used to check whether the data was missing randomly. Values for respondents who answered ‘no’, ‘yes’ and missing on these items of the TFI were compared on the following variables: Age, gender and TOPICS-MDS data on frailty.

As a second step to analyse the potential consequences of the missing data on items 3 through 8 on the TFI, it was decided to impute the data to compare the results of the original data and the imputed data. Variables used to create the imputed data file were: 15 TFI items, age (continuous), gender (male/female), marital status (married/other) and loneliness (not, moderate, serious, very serious). Marital status and loneliness were dichotomized respectively categorized to lower the number of parameters to be able to create the imputed data. The highest percentage of missing was 25.8, which resulted in 26 imputations with 100 iterations.

A multiple regression analysis was performed to determine which variables contribute to the total score of frailty. The following variables were used in this analysis: age, gender, marital status, living situation, income, health perception, number of conditions, ADL problems, home care and informal care. Dummy variables were created for categorical variables.

To assess significant differences between the different domains of frailty a one way ANOVA test was used for the continuous variables. For the variables ADL, number of conditions, quality of life and loneliness, the variances within the different groups were not equal according to Levene’s test of homogeneity of variances. However, because of the large sample size, Bonferroni post hoc analyses were used to identify the significant differences between the subgroups.

Chi square tests were done for the dichotomous and categorical variables. Post hoc analyses, using the standardized residuals, were done to show whether the observed results per subgroup differed from the expected. When the standardized residual differed more than 2 or − 2 from the expected, it was designated as different from the expected [31]. *P*-values lower than 0.05 were considered as significant.

## Results

### Missing value analysis

The missing value analysis showed that the respondents with missing values on items three up to eight were more often female and their mean age lied between the mean age of the respondents who answered ‘yes’ and ‘no’ on those items. Also, the frailty score of the TOPICS-MDS was calculated to compare the results with the TFI. For respondents who had missing values for TFI items three up to eight, it was assessed whether they were frail according to the frailty index of TOPICS-MDS 0.20. On all six items, there were no substantial differences on the percentage of frail respondents according to the TOPICS-MDS 0.20. The Little’s Missing Completely at Random (MCAR) Test showed that the missing values were at random (*p* = .685).

Additional file 2 shows the scores on the items of the TFI for the original and imputed data. Also, the different domains of frailty are shown: physical, psychological, social and the possible combinations of these. For the imputed data, the scores on the physical domain of frailty increased substantially when compared to the original dataset and as a result of that the total score on frailty also increased. When relating these results to the outcomes of the missing value analysis the imputed data appear to overestimate the scores on frailty and therefore also the number of frail respondents. In addition, Additional file 2 shows that physical frailty would increase when using the imputed data, but still social frailty and frailty on all domains would remain the most often identified forms of frailty. Based on these findings, it was decided to use the original dataset for analyses, to prevent the risk of overestimating the outcomes on the physical domain and frailty in total.

### Background characteristics

The mean age of the 1768 respondents was 78.7 years (SD = 6.4) and a large proportion was female (69.1%). More than half of the respondents was widowed (51.1%) followed by the married respondents (32.4%). The majority of the respondents lived independently and alone (65.1%). Background characteristics are shown in Table 2, including an overview of the different domains of frailty.

### Frailty

In total, 51.6% of the respondents was frail according to the TFI. Table 1 shows data of the original dataset. 68.9% was frail on one or multiple domains and 31.1% was not frail at all. The type of frailty most often present among the respondents was social frailty (18.4%) and frailty on all domains (10.3%).

**Table 1:** Scores and missing values on the frailty items in the original data

Item	Yes (%)	No (%)	Sometimes (%)	Missing (%)
Physical frailty				
1 Poor physical health	1010 (57.1)	663 (37.5)*	NA	95 (5.4)
2 Unexplained weight loss	**161 (9.1)**	1506 (85.2)	NA	101 (5.7)
3 Difficulty in walking	**800 (45.2)**	725 (41.0)	NA	243 (13.7)
4 Difficulty maintaining balance	**517 (29.2)**	853 (48.2)	NA	398 (22.5)
5 Poor hearing	**465 (26.3)**	906 (51.2)	NA	397 (22.5)
6 Poor vision	**331 (18.7)**	980 (55.4)	NA	457 (25.8)
7 Strength in hands	**544 (30.8)**	840 (47.5)	NA	384 (21.7)
8 Physical tiredness	**777 (43.9)**	694 (39.3)	NA	297 (16.8)
Psychological frailty				
9 Problems with memory	**114 (6.4)**	982 (55.5)	609 (34.4)	63 (3.6)
10 Feeling down	**152 (8.6)**	873 (49.4)	**670 (37.9)**	73 (4.1)
11 Feeling nervous or anxious	**136 (7.7)**	995 (56.3)	**577 (32.6)**	60 (3.4)
12 Able to cope with problems	1426 (80.7)	**263 (14.9)**	NA	79 (4.5)
Social frailty				
13 Living alone	**1138 (64.4)**	585 (33.1)	NA	45 (2.5)
14 Social relations	**393 (22.2)**	758 (42.9)	**565 (32.0)**	52 (2.9)
15 Social support	1406 (79.5)	**285 (16.1)**	NA	77 (4.4)

*The bold items contribute to frailty

The results of the multivariate analysis of the health aspects show that the following aspects significantly contribute to the total score of the TFI: living independent alone (+), health perception very good/excellent (−), health perception good (−), home care (+), ADL score (+), number of conditions (+).

Next, statistical analyses were performed to test for potential differences between the health and wellbeing variables [age, gender, marital status, living situation, income, health perception, number of conditions, ADL problems, home care, informal care, quality of life, loneliness, going outside, meeting the children and grandchildren, meeting the family and in-laws, meeting friends, meeting neighbours] and the domains of frailty. All tested variables (shown in Tables 2 and 3), except for income, showed significant differences for either the expected values or differences between the subgroups of frailty.

**Table 2:** Analyses on background characteristics for different domains of frailty

Background characteristics	Total *n* = 1768 Respondents (%/SD)	Missing (%)	0 *n* = 550 (31.1%)	1 *n* = 147 (8.3%)	2 *n* = 132 (7.5%)	3 *n* = 325 (18.4%)	4 *n* = 107 (6.1%)	5 *n* = 100 (5.7%)	6 *n* = 225 (12.7%)	7 *n* = 182 (10.3%)
Mean Age (SD)** Range: 65–99	78.7 (6.4)	35 (2.0)	78.19 (6.2)	80.86 (6.2)	78.22 (6.0)	78.83 (6.3)	78.33 (6.7)	80.44 (6.7)	77.26 (6.3)	79.67 (6.8)
Posthoc analysis shows significant differences for: 0–1**, 0–5*, 1–2*, 1–3*, 3–4**, 1–6*, 5–7*										
65–74*	485 (27.4)		153 (33.4)	22 (4.8)	33 (7.2)	81 (17.7)	34 (7.4)	17 (3.7)	78 (17.0)	40 (8.7)
75–79	464 (26.3)		153 (33.0)	36 (7.8)	39 (8.4)	84 (18.1)	23 (5.0)	26 (5.6)	55 (11.9)	48 (10.3)
80+	811 (45.9)		232 (28.6)	87 (10.7)	57 (7.0)	153 (18.9)	48 (5.9)	54 (6.7)	87 (10.7)	93 (11.5)
Posthoc analysis shows differences compared to the expected for: 65–74: 1 (−), 6 (+), 80+: 1 (+)										
Gender (female)**	1221 (69.1)	0	310 (56.4)	86 (58.5)	83 (62.9)	267 (82.8)	64 (59.8)	81 (81.0)	180 (80.0)	151 (83.0)
Posthoc analysis shows differences compared to the expected for: male: 0, female: 3,6,7										
Marital status**		6 (0.3)								
Widowed	904 (51.1)		201 (36.5)	53 (36.1)	43 (32.6)	234 (72.0)	23 (21.5)	70 (70.0)	158 (70.2)	122 (67.0)
Married	573 (32.4)		265 (48.2)	86 (58.5)	76 (57.6)	20 (6.2)	73 (68.2)	6 (6.0)	22 (9.8)	25 (13.7)
Divorced	179 (10.1)		49 (8.9)	5 (3.4)	9 (6.8)	41 (12.6)	8 (7.6)	14 (14.0)	27 (12.0)	26 (14.3)
Other	106 (5.9)		33 (6.0)	3 (2.1)	4 (3.0)	28 (8.6)	3 (2.9)	9 (9.0)	18 (8.0)	8 (4.3)
Posthoc analysis shows differences compared to the expected for: widowed: 0 (−), 1 (−), 2 (−), 3 (+), 4 (−), 5 (+), 6 (+), 7 (+)| Married: 0 (+), 1 (+), 2 (+), 3 (−), 4 (+), 5 (−), 6 (−), 7(−) | Divorced: 1 (−) | other 1 (−)										
Living situation**^a^		12 (0.7)								
Independent *alone*	1151 (65.1)		262 (47.6)	56 (38.1)	50 (37.9)	309 (95.1)	31 (29.0)	93 (93.0)	201 (89.3)	149 (81.9)
Independent *with others*	579 (32.7)		280 (50.9)	85 (57.8)	79 (59.8)	14 (4.3)	76 (71.0)	5 (5.0)	19 (8.4)	21 (11.5)
Independent living *with possible assistance*	25 (1.4)		5 (0.9)	5 (3.4)	1 (0.8)	0	0	2 (2.0)	3 (1.3)	10 (5.5)
Posthoc analysis shows differences compared to the expected for: Independent alone: 0 (−), 1 (−), 2 (−), 3 (+), 4 (−), 5 (+), 6 (+), 7(+) | Independent with others: 0 (+), 1 (+), 2 (+), 3 (−), 4 (+), 5 (−), 6 (−), 7 (−)										
Income (Lower than €1200 net per month)	598 (33.8)	137 (7.7)	160 (29.1)	44 (29.9)	40 (30.3)	120 (36.9)	43 (40.2)	40 (40)	76 (33.8)	75 (41.2)
Health perception**		8 (0.5)								
Excellent/very good	219 (12.4)		121 (22)	2 (1.4)	11 (15.6)	62 (19)	0	3 (3)	19 (8.4)	1 (0.5)
Good	611 (34.6)		247 (44.9)	36 (24.5)	57 (43.2)	145 (44.6)	11 (10.3)	17 (17)	81 (36)	17 (9.3)
Reasonable/bad	930 (52.6)		178 (32.4)	108 (73.5)	64 (48.5)	117 (36)	95 (88.8)	80 (80)	124 (55.2)	164 (90.1)
Posthoc analysis shows differences compared to the expected for: excellent very good: 0 (+), 1 (−), 3 (−), 4 (−), 5 (−), 7 (−) | good: 0 (+), 1 (−), 3 (+), 4 (−), 5 (−), 7 (−) | reasonable/bad: 0 (−), 1 (+), 3 (−), 4 (+), 5 (+), 7 (+)										
Number of conditions** (Range: 0–11)	2.61 (1.85)	Unknown	1.9 (1.4)	3.6 (1.7)	2.3 (1.5)	1.9 (1.3)	4.1 (1.8)	3.6 (1.8)	2.3 (1.6)	4.5 (2.0)
Posthoc analysis shows significant differences for: 0–1**, 0–4**, 0–5**, 0–6*, 0–7**, 1–2**, 1–3**, 1–6**, 1–7**, 2–4**, 2–5**, 2–7**, 3–4**, 3–5**, 3–6*, 3–7**, 4–6**, 5–6**, 5–7**, 6–7**										
ADL problems (Range: 0–15)**	2.2 (2.5)	51 (2.9)	1.08 (1.7)	4.0 (3.2)	1.6 (2.0)	1.3 (1.5)	4.9 (3.3)	3.5 (2.1)	1.6 (1.7)	4.1 (2.6)
Posthoc analysis shows significant differences for: 0–1*, 0–4**, 0–5**, 0–7**, 1–2**, 1–3**, 1–4*, 1–6**, 2–4**, 2–5**, 2–7**, 3–4**, 3–5**, 3–7**, 4–5**,4–6**, 5–6**, 6–7**										
Help or assistance at home										
Home care**	762 (43.1)	23 (1.4)	145 (26.4)	82 (55.8)	43 (32.6)	129 (39.7)	66 (61.7)	71 (71.0)	90 (40.0)	136 (74.7)
Posthoc analysis shows differences compared to the expected for: 0 (−), 1 (+), 4 (+), 5 (+), 7 (+)										
Informal care**	360 (20.4)	28 (2.9)	74 (13.5)	63 (42.9)	25 (18.9)	35 (10.8)	49 (45.8)	25 (25)	22 (9.8)	67 (36.8)
Posthoc analysis shows differences compared to the expected for: 0 (−), 1 (+), 3 (−), 4 (+), 6 (−), 7 (+)										

Not frail for all domains (0), Physical frail (1), psychological frail (2), social frail (3), Physical and psychological (4), Physical and social (5), Psychological and social (6), Frail on all three domains (7)

^a^ The group ‘Independent living with possible assistance’ has been left out of the analysis because the group was too small

*Analysis shows a significance between 0,05-0,001

**Analysis shows a significance lower than 0,00

**Table 3:** Analyses on wellbeing aspects for different domains of frailty

Wellbeing	Total *n* = 1768 Respondents (%/SD)	Missing (%)	0 *n* = 550 (31.1%)	1 *n* = 147 (8.3%)	2 *n* = 132 (7.5%)	3 *n* = 325 (18.4%)	4 *n* = 107 (6.1%)	5 *n* = 100 (5.7%)	6 *n* = 225 (12.7%)	7 *n* = 182 (10.3%)
Quality of life										
Grade (SD)^b^	7.3 (1.2)	30 (1.7)	7.9 (0.9)	7.3 (1.1)	7.2 (1.2)	7.6 (1.0)	6.5 (1.0)	7.1 (0.9)	6.8 (1.2)	6.2 (1.2)
Posthoc analysis shows significant differences for: 0–1^b^, 0–2^b^, 0–3^b^, 0–4^b^, 0–5^b^, 0–6^b^, 0–7^b^, 1–4^b^, 1–6^b^, 1–7^b^, 2–3^a^, 2–4^b^, 2–6^a^, 2–7^b^, 3–4^b^, 3–5^b^, 3–6^b^, 3–7^b^, 4–6^a^, 5–7^b^										
Compared to one year ago^b^		26 (1.5)								
Much better	71 (4.0)		21 (3.8)	6 (4.1)	2 (1.5)	26 (8.0)	1 (0.9)	5 (5.)	7 (3.1)	3 (1.6)
Little better	127 (7.2)		29 (5.3)	8 (5.4)	10 (7.6)	25 (7.7)	8 (7.5)	5 (5)	23 (10.2)	19 (10.4)
the same	1105 (62.5)		430 (78.2)	80 (54.4)	81 (61.4)	226 (69.5)	42 (39.3)	57 (57)	134 (59.6)	55 (30.2)
Little worse	374 (21.2)		60 (10.9)	45 (30.6)	35 (26.5)	4 (13.5)	40 (37.4)	27 (27)	45 (20.0)	78 (42.9)
Much worse	65 (3.7)		2 (0.4)	6 (4.1)	3 (2.3)	1 (0.3)	14 (13.1)	5 (5)	8 (3.6)	26 (14.3)
Posthoc analysis shows differences compared to the expected for: much better: 3 (+) | the same: 0 (+), 4 (−), 7(−) | little worse: 0 (−), 1 (+), 3 (−), 4 (+), 7 (+) | much worse: 0 (−), 3 (−), 4 (+), 7 (+)										
Loneliness^b^ Range: 0–11	3.9 (3.5)	217 (12.3)	1.7 (2.1)	2.3 (2.6)	3.1 (3.1)	4.2 (3.2)	4 (3.7)	5.2 (3.2)	6.9 (3.7)	7.1 (3)
Posthoc analysis shows significant differences for: 0–2^b^, 0–3^b^, 0–4^b^, 0–5^b^, 0–6^b^, 0–7^b^, 1–3^b^, 1–4^b^, 1–5^b^, 1–6^b^, 1–7^b^, 2–3^a^, 2–4^b^, 2–7^b^, 3–6^b^, 3–7^b^, 4–6^b^, 4–7^b^, 5–6^b^, 5–7^b^										
Going outside^b^		57 (3.3)								
Daily	917 (51.9)		360 (65.5)	49 (33.3)	65 (49.2)	181 (55.7)	39 (36.4)	35 (35)	132 (58.7)	56 (30.8)
Few times per week	619 (35.0)		128 (23.3)	67 (45.6)	55 (41.7)	128 (39.4)	45 (42.1)	49 (49)	71 (31.6)	76 (41.8)
Few times per month	113 (6.4)		17 (3.1)	21 (14.3)	7 (5.3)	8 (2.5)	12 (11.2)	8 (8)	12 (5.3)	28 (15.4)
Less than once a month	62 (3.5)		7 (1.3)	9 (6.1)	3 (2.3)	1 (0.3)	10 (9.3)	7 (7)	6 (2.7)	19 (10.4)
Posthoc analysis shows differences compared to the expected for: daily: 0 (+), 1 (−), 4 (−), 5 (−), 7 (−) | few times per week: 0 (−), 1 (+), 5 (+) | few times per month: 0 (−), 1 (+), 3 (−), 7 (+) | less than once a month: 0 (−), 4 (+), 7 (+)										
How often do you meet …										
Children or grandchildren^a^		228 (12.9)								
Every week	771 (43.6)		256 (46.5)	78 (53.1)	53 (40.2)	136 (41.8)	54 (50.5)	37 (37)	84 (37.3)	73 (40.1)
Once or twice per month	399 (22.6)		119 (13.5)	31 (21)	38 (18.8)	72 (22.1)	22 (20.6)	28 (28)	47 (20.9)	45 (14.7)
Less/seldom	161 (9.1)		33 (4.2)	12 (8.1)	13 (9.8)	32 (9.9)	15 (14)	10 (10)	27 (12)	19 (10.4)
Never	57 (3.2)		13 (2.4)	5 (3.4)	3 (2.3)	10 (3.1)	5 (4.7)	4 (4)	9 (4)	8 (4.4)
Not applicable	152 (8.6)		42 (7.6)	8 (5.4)	7 (5.3)	33 (10.2)	8 (7.5)	15 (15)	29 (12.9)	10 (5.5)
Posthoc analysis shows differences compared to the expected for: less/seldom: 0 (−) | not applicable: 6 (+)										
Family/in-laws^b^		314 (17.8)								
Every week	289 (16.3)		109 (19.8)	25 (17)	13 (9.8)	62 (19.1)	20 (18.7)	12 (12)	29 (12.9)	19 (10.4)
Once or twice per month	371 (21.0)		122 (22.2)	39 (26.6)	35 (26.5)	58 (17.8)	22 (20.5)	19 (19)	39 (17.3)	37 (20.3)
Less/seldom	410 (23.2)		99 (18)	35 (23.8)	35 (27.1)	72 (22.2)	28 (26.2)	30 (30)	63 (28)	48 (26.4)
Never	177 (10.0)		44 (8)	10 (6.8)	4 (3)	31 (9.5)	14 (13.1)	17 (17)	30 (13.3)	27 (14.8)
Not applicable	207 (11.7)		63 (11.5)	25 (17)	17 (12.9)	40 (12.3)	13 (12.1)	9 (9)	21 (9.3)	19 (10.4)
Posthoc analysis shows differences compared to the expected for: every week: 0 (+), 7 (−) | less /seldom: 0 (−) | never: 2 (−), 5 (+), 7 (+)										
Friends^b^		172 (9.7)								
Every week	728 (41.2)		259 (47.1)	67 (45.6)	50 (37.9)	143 (44)	39 (36.4)	33 (33)	90 (40)	47 (25.8)
Once or twice per month	506 (28.6)		147 (26.8)	44 (30)	42 (31.8)	94 (28.9)	33 (30.9)	31 (31)	62 (27.1)	53 (29.1)
Less/seldom	264 (15.0)		59 (10.8)	16 (10.9)	21 (15.9)	35 (10.7)	27 (23.4)	27 (27)	38 (16.8)	41 (22.5)
Never	64 (3.6)		7 (1.3)	5 (3.4)	3 (2.3)	13 (4)	3 (2.8)	6 (6)	8 (3.6)	19 (10.4)
Not applicable	34 (1.9)		(71.3)	6 (4.1)	2 (1.5)	6 (1.8)	3 (2.8)	0	4 (1.8)	6 (6.3)
Posthoc analysis shows differences compared to the expected for: every week: 0 (+), 7 (−) | less/seldom: 0 (−), 4 (+), 5 (+) | never: 0 (−), 7 (+)										
Neighbours^b^		144 (8.1)								
Every week	1006 (56.9)		375 (68.2)	87 (59.2)	74 (56.1)	181 (55.7)	51 (47.7)	47 (47)	116 (51.6)	75 (41.2)
Once or twice per month	298 (16.8)		67 (12.2)	27 (18.4)	24 (18.1)	67 (20.3)	22 (20.6)	20 (20)	24 (15.1)	38 (20.9)
Less/seldom	218 (12.3)		32 (5.6)	17 (11.6)	17 (12.9)	37 (11.4)	23 21.5)	17 (17)	43 (19.1)	32 (17.6)
Never	80 (4.5)		8 (1.5)	7 (4.8)	2 (1.5)	8 (2.5)	8 (7.5)	11 (11)	14 (6.2)	22 (12.1)
Not applicable	22 (1.2)		8 (1.5)	2 (1.4)	1 (0.8)	5 (1.5)	1 (0.9)	2 (2)	1 (0.4)	2 (1.1)
Posthoc analysis shows differences compared to the expected for: every week: 0 (+), 7 (−) | once or twice per month: 0 (−) | less/seldom: 0 (−), 4 (+), 5 (+), 7 (+) | never: 0 (−), 5 (+), 7 (+)										

Not frail for all domains (0), Physical frail (1), psychological frail (2), social frail (3), Physical and psychological (4), Physical and social (5), Psychological and social (6), Frail on all three domains (7)

^a^ Analysis shows a significance between 0,05-0,001

^b^ Analysis shows a significance lower than 0,00

The overall analysis on age groups and the domains of frailty showed a significant difference for the different domains of frailty (*p* = 0.001). Physical frailty was more often present in the higher age group (80+) and less in the lower age group (65–74). This increase by age was not seen for other domains of frailty.

The overall analysis for gender in relation to the different domains of frailty also showed a significant difference (*p* < 0.001); the physically, psychologically-socially and all-domains frail respondents were more often female (82.8, 80.0 and 83.0%) and the non-frail were more often male (43.6%). Related to this, the marital status also significantly differed for the different domains of frailty (*p* < 0.001). The percentage widowed respondents was higher for respondents who were frail on the domains social, physical-social, psychological-social and on all domains.

### Health and care

There was a significant difference between the categories of frailty and the perceived health by the respondents (*p* < 0.001). The health perception for the socially frail was higher and the health perception for the physically, physically-psychologically, physically-socially and all-domains frail respondents was lower.

The overall analysis shows significant differences for the mean number of conditions (*p* < 0.001) and the mean number of ADL problems (*p* < 0.001) between the subgroups of frailty. The largest differences for the number of conditions was seen between the subgroups non-frail versus frail on all domains (MD = -2.66, *p* < 0.001) and socially frail versus frail on all domains (MD = -2.7, p < 0.001).

The largest mean difference for ADL problems was found for non-frail versus physically-psychologically frail (MD = − 3.8, *p* < 0.001), socially versus physically-psychologically frail (MD = -3.5, p < 0.001), physically-psychologically frail versus psychologically-socially frail (MD = 3.3, p < 0.001), physically-psychologically frail versus psychologically frail (MD = 3.3, p < 0.001). This shows that physical frailty was more associated with the number of ADL problems compared to the other domains of frailty.

There was a significant difference between the categories of frailty and the use of home care (*p* < 0.001). The respondents who were frail on the domains physical, physical-psychological, physical-social and on all domains used this type of care significantly more than expected.

In total, 360 respondents (20.4%) had an informal care provider. There was a significant difference for the overall analysis of having an informal care provider and the frailty categories (*p* < 0.001). Respondents who were frail on the domains physical, physical-psychological and all domains had an informal care provider more often. The non-frail, socially frail and psychologically-socially frail respondents had an informal care provider less often.

### Wellbeing

Table 3 shows aspects of wellbeing for the different domains of frailty. The overall test for quality of life shows significant differences between the categories of frailty (*p* < 0.001). Quality of life was the highest for the non-frail (mean = 7.9, SD = 0.87) and lowest for the respondents who were frail on all domains (mean = 6.2, SD = 1.2) (MD = -1.72, p < 0.001). All different domains have a significantly lower quality of life grade compared to the non-frail respondents.

The total loneliness instrument was completed by 1551 (87.7%) respondents. 22.1% scored 0 points on the loneliness instrument and 5.2% scored the total 11 points. The mean score for loneliness was 3.86 (SD = 3.5). Respondents who were frail on all domains had the highest average score on loneliness (7.1, SD = 3.0), followed by psychologically frail and socially frail respondents (6.9, SD = 3.4) and physically-socially frail respondents (5.2, SD = 3.2). Loneliness was lowest for the non-frail (mean = 1.7, SD = 3.0). The overall analysis showed a significant difference between these subgroups (*p* < 0.001).

The number of times respondents went outside was analysed and the overall test shows that there was a significant difference for the different domains of frailty (p < 0.001). Respondents who were physically-psychologically frail and respondents who were frail on all domains scored significantly higher on the category “coming outside less than once a month”.

The overall analysis for the different types of contact all show significant differences for the different domains of frailty; namely contact with (grand)children (*p* = 0.035), family/in-laws (*p* < 0.001), friends (*p* < 0.001) and neighbours (*p* < 0.001).

The psychologically-socially frail respondents more often do not have children and grandchildren compared to all other domains.

## Discussion

Results of this study complement the existing literature on frailty since it distinguishes the different domains of frailty and relate it to potential variables. More older adults will have to live independently and be self-sufficient. Therefore, it is important to offer sufficient care and support to adequately assist the independent living situation. Assessing frailty for the different domains provides insight into different needs, which is important for policymakers and care providers.

Our results show that care and wellbeing characteristics are related to the domains of frailty. The proportion of frailty increases when looking at the different domains compared to the total TFI score. These results support the results of a study by Oostrom et al. in 2017; they state that it is important to consider the different domains since this helps to identify different groups of frail people, and therefore enables professionals to provide tailored care and support [32]. In our study 68.9% of the respondents was frail on one or multiple domains. This proportion was higher compared to the proportion of frail respondents on the total TFI score (51.6%) because the score for social (two or three) or psychological frailty (two, three or four) was lower than the overall score for frailty (five). Implicating that it is important to not only look at the total frailty score but to take the different domains into account as well, with a focus on social frailty (18.4%) and frailty on all three domains (10.3%), since these were most often present. A study by Gobbens and van Assen also emphasizes the necessity to use a multidimensional approach. They implicate that problems with walking, feeling down and a lack of social support are important factors that influence the quality of life in older adults, and quality of life in older persons is associated with frailty [33].

Physical frailty is the only domain that showed an association with the different age groups and increases with age. Other research supports this finding [34]. This implies that psychological and social frailty may have a different onset and pattern of development than physical frailty and support may be needed at a specific time in the lives of the older adults. Social frailty may, for example, occur after a big life event such as the loss of a spouse. ‘Living alone’ is one of the items for social frailty and may explain this association. Different studies indicate that frailty increases with age [11, 35]. However, our study, that of Gobbens et al. [28] and van Assen et al. [34] suggest that this is mainly explained by physical frailty. However, as discussed in the introduction, health is no longer just the absence of diseases, it is seen as a state of complete wellbeing on different domains. Frailty in one of the three domains may jeopardize the health status of an older adult and therefore it is important to include all three domains.

Also, physical frailty and combinations with physical frailty show associations mainly on the health aspects. When offering care and support to the frail older adults, this is an important insight to consider since the needs for psychologically and socially frail older adults may be less focussed on care than for those who are only physically frail. For physical frailty it seems more likely that care is needed to provide support, for example in the ADL-activities. For the socially frail, it can be hypothesized that social support, for example in the form of activities and contacts by a volunteer, is needed to increase their wellbeing.

The proportion of frailty based on the total TFI score (five or higher) was high (51.6%). This possibly could be explained by the fact that respondents already were a member of a welfare organization and therefore in need of more support. However, when looking at the mean age (78.7 SD = 6.4) the proportion of frailty closely matches that of the SCP study [14] for respondents of 80 years and older (50%). A slightly larger sample of female respondents (69.1%) could explain the remaining difference, since women tend to be frail more often than men [36]; in 2012 65% of people over 80 was female in the Netherlands [37].

### Strengths and limitations

A large amount (*n* = 1768) of older adults was reached for this baseline measurement, even though the response rate was only moderate with 35,1%. The questionnaire contained many topics and many variables were tested to gain an overall view of the individual status of the respondent. Also, where possible, validated tools were used to measure the outcomes.

Unfortunately, data on the TFI items showed a significant number of missing values. A possible explanation could be that items three up to eight of the TFI were presented in a different way than the other items and respondents only checked a box when their answer was ‘yes’. In addition, a large part of the respondents filled in the questionnaire by themselves, missing data was more often present for those respondents. Data was therefore imputed and compared to the original data which showed an increase in physical frailty and domains combined with physical frailty. Because of this and the results of the missing value analysis, it was decided to analyse the data using the original data to prevent an overestimation of frailty. Nevertheless, it should be taken into account that the original data could have shown an underestimation for the proportion of frailty and especially on the domain of physical frailty.

Respondents were recruited via a welfare organisation in the province of North-Holland in the Netherlands. This sample may be specific since these older adults are already registered with a welfare organization, and may be in need of welfare and support. However, it is unlikely that this will affect the main message of this paper, being that it is important to look at the different domains of frailty to provide tailored care and support instead of looking at frailty in general. Nevertheless, when considering generalising the data these differences should be taken into account. However, the data and numbers of frailty in the current study generally match the data of the SCP study in which national data of the Netherlands was used [14].

Reasons for non-responding are not available to the researchers. For privacy reasons the welfare organizations contacted the older adults instead of the researchers and reasons for not participating could not be identified. This may have led to some information bias since reasons and characteristics of the non-responders are unknown.

### Recommendations

With the rising number of frail independent living older adults and policy focussed on self-sufficiency and independent living it is important to assess the frail older adults and their care and support needs on an individual basis. Data of this study show that a distinction between the different domains of frailty may provide valuable information about the characteristics for these different domains and what type of care or support is needed in each domain respectively. Our findings support other studies which emphasize the need to use multidimensional tools to improve wellbeing of the older adults and the quality of their care and support [32, 33]. Further research is recommended to assess specific care and/or support needs and wishes for the different domains.

Our results may contribute to configure the ageing in place challenges. Since ageing in place is an widely adopted concept, it is important to assist older adults in living independently. Our study shows that older adults have limitations and therefore needs on different domains. Local governments and care providers will be better able to provide tailored support when they have insight into the specific domains of frailty. Based on the retrieved data, policy can be formulated. However, it will be an important challenge for municipalities and other local governments to incorporate the social and physical domain, therefore they have to integrate their activities with those of care and welfare organizations, clients and housing cooperatives in order to appropriately support the older adults in living independent as long as possible while maintaining health and wellbeing.

## Conclusion

Distinguishing the three different domains of frailty provides information which is valuable for policymakers and care providers, to anticipate to the increasing number of independent living older adults and deliver them tailored care and support to contribute to their independent living situation and wellbeing.

## Additional files

Additional file 1: Complementary questions. (DOCX 15 kb)

Additional file 2: Table with frailty scores for original and imputed data. (DOCX 15 kb)

## Abbreviations

ADL: Activities of daily living
MCAR: Missing Completely at Random
MD: Mean difference
SCP: Netherlands Institute for Social Research
TFI: Tilburg Frailty Indicator
Topics-MDS: The older persons and informal caregivers survey minimum dataset
VUmc: Vrije Universiteit (Medical Center)
WHO: World Health Organization
ZonMw: Netherlands Organization for Health Research and Development
