# Supplementary material for: A cross sectional study on the different domains of frailty for independent living older adults
Source: BMC Geriatr. 2019 Mar 1;19:61. doi: 10.1186/s12877-019-1077-3 (PMC6397452; doi:10.1186/s12877-019-1077-3)
Supplement: Supplementary file 1 — Complementary questions. (DOCX 15 kb) [file 12877_2019_1077_MOESM1_ESM.docx]

**Complementary questions**

1/15 TFI, see manuscript for reference

16. Do you have a long term disease, handicap or condition?

1. Yes
2. No

17. Can you move independently

1. Yes
2. No
3. Hardly

18. Can you climb up and down the stairs

1. Yes
2. No
3. Hardly
4. NA

19. Can you move yourself outside

1. Yes
2. No
3. Hardly

20. Do you have and use a car

1. Yes
2. No

21. Do you use public transport

1. Yes
2. No

22. Do you use a taxi, or local busses on demand

1. Yes
2. No

23. in general, how often do you go outside your house or go somewhere

1. Daily
2. Few times per week
3. Few times per month
4. Less than once a month

24. How often do you meet the following people?

| *Contact* | ***Every week*** | ***Twice a month*** | ***Once a month*** | ***less*** | ***rarely*** | ***never*** | ***NA*** |
| --- | --- | --- | --- | --- | --- | --- | --- |
| Children or grand children | ❑ | ❑ | ❑ | ❑ | ❑ | ❑ | ❑ |
| Family or in-laws | ❑ | ❑ | ❑ | ❑ | ❑ | ❑ | ❑ |
| Friends | ❑ | ❑ | ❑ | ❑ | ❑ | ❑ | ❑ |
| Neighbours | ❑ | ❑ | ❑ | ❑ | ❑ | ❑ | ❑ |

25. Are you satisfied with how often you meet other people

1. Yes
2. No, I would like to meet the people I know more often
3. No, I would like to meet new people
4. I would like to meet less people

26-36 Loneliness scale de Jong-Gierveld, see manuscript for reference

37. When you live alone, is your nett income lower than €1200 per month

1. Yes
2. No

38. When you live with other people, is your nett income lower than €1600 per month

1. Yes
2. No

39. Do you have an informal care provider

1. Yes
2. No
