# Supplementary material for: A cross sectional study on the different domains of frailty for independent living older adults
Source: BMC Geriatr. 2019 Mar 1;19:61. doi: 10.1186/s12877-019-1077-3 (PMC6397452; doi:10.1186/s12877-019-1077-3)
Supplement: Supplementary file 2 — Table with frailty scores for original and imputed data. (DOCX 15 kb) [file 12877_2019_1077_MOESM2_ESM.docx]

**Additional file 2:** Table with frailty scores for original and imputed data.

|  | **Original data** | |  | **Imputed data** | |
| --- | --- | --- | --- | --- | --- |
| **Items TFI** | Contributes to frailty | Does not contribute to frailty |  | Contributes to frailty | Does not contribute to frailty |
| TFI1 | 37.5% | 62.5% |  | 39.5% | 60.3% |
| TFI2 | 9.1% | 90.9% |  | 9.5% | 90.2% |
| TFI3 | **45.2%** | 54.8% |  | **53.2%** | 46.2% |
| TFI4 | **29.2%** | 70.8% |  | **40.7%** | 58.5% |
| TFI5 | **26.3%** | 73.7% |  | **36.2%** | 63.0% |
| TFI6 | **18.7%** | 81.3% |  | **28.5%** | 70.5% |
| TFI7 | **30.8%** | 69.2% |  | **42.7%** | 56.5% |
| TFI8 | **43.9%** | 56.1% |  | **54.2%** | 45.2% |
| TFI9 | 6.4% | 93.6% |  | 6.6% | 93.2% |
| TFI10 | 46.5% | 53.5% |  | 48.4% | 51.5% |
| TFI11 | 40.3% | 59.7% |  | 41.7% | 58.2% |
| TFI12 | 14.9% | 85.1% |  | 15.4% | 84.4% |
| TFI13 | 64.4% | 35.6% |  | 65.9% | 34.0% |
| TFI14 | 54.2% | 45.8% |  | 55.7% | 44.2% |
| TFI15 | 16.1% | 83.9% |  | 16.9% | 83.0% |
| Total TFI score (SD) | 4.84 (3.04) | |  | 5.56 (3.25) | |
| Frail | 51.6% | |  | 58.4% | |
| Not frail for all domains | 31.1% | |  | 24.9% | |
| Physical frailty | 8.3% | |  | 11.9% | |
| Psychological frailty | 7.5% | |  | 6.0% | |
| Social frailty | 18.4% | |  | 14.6% | |
| Physical and psychological | 6.1% | |  | 8.0% | |
| Physical and social | 5.7% | |  | 9.5% | |
| Psychological and social | 12.7% | |  | 9.5% | |
| Physical, psychological and social | 10.3% | |  | 14.1% | |
